# Mapping small mammal optimal habitats using satellite-derived proxy variables and species distribution models

**DOI:** 10.1371/journal.pone.0289209

**Published:** 2023-08-17

**Authors:** Christopher Marston, Francis Raoul, Clare Rowland, Jean-Pierre Quéré, Xiaohui Feng, Renyong Lin, Patrick Giraudoux

**Affiliations:** 1 UK Centre for Ecology and Hydrology, Lancaster, United Kingdom; 2 Department of Chrono-Environment, University of Bourgogne Franche-Comte/CNRS, Besançon, France; 3 Centre de Biologie et Gestion des Populations (INRAE/IRD/Cirad/Montpellier SupAgro), Campus International de Baillarguet, Montferrier-sur-Lez Cedex, France; 4 WHO-Collaborating Centre for Prevention and Care Management of Echinococcosis, The First Affiliated Hospital of Xinjiang Medical University, Urumqi, Xinjiang, China; 5 Yunnan University of Finance and Economics, Kunming, China; Universidade Federal de Uberlandia, BRAZIL

## Abstract

Small mammal species play an important role influencing vegetation primary productivity and plant species composition, seed dispersal, soil structure, and as predator and/or prey species. Species which experience population dynamics cycles can, at high population phases, heavily impact agricultural sectors and promote rodent-borne disease transmission. To better understand the drivers behind small mammal distributions and abundances, and how these differ for individual species, it is necessary to characterise landscape variables important for the life cycles of the species in question. In this study, a suite of Earth observation derived metrics quantifying landscape characteristics and dynamics, and in-situ small mammal trapline and transect survey data, are used to generate random forest species distribution models for nine small mammal species for study sites in Narati, China and Sary Mogul, Kyrgyzstan. These species distribution models identify the important landscape proxy variables driving species abundance and distributions, in turn identifying the optimal conditions for each species. The observed relationships differed between species, with the number of landscape proxy variables identified as important for each species ranging from 3 for *Microtus gregalis* at Sary Mogul, to 26 for *Ellobius tancrei* at Narati. Results indicate that grasslands were predicted to hold higher abundances of *Microtus obscurus*, *E*. *tancrei* and *Marmota baibacina*, forest areas hold higher abundances of *Myodes centralis* and *Sorex asper*, with mixed forest—grassland boundary areas and areas close to watercourses predicted to hold higher abundances of *Apodemus uralensis* and *Sicista tianshanica*. Localised variability in vegetation and wetness conditions, as well as presence of certain habitat types, are also shown to influence these small mammal species abundances. Predictive application of the Random Forest (RF) models identified spatial hot-spots of high abundance, with model validation producing R^2^ values between 0.670 for *M*. *gregalis* transect data at Sary Mogul to 0.939 for *E*. *tancrei* transect data at Narati. This enhances previous work whereby optimal habitat was defined simply as presence of a given land cover type, and instead defines optimal habitat via a combination of important landscape dynamic variables, moving from a human-defined to species-defined perspective of optimal habitat. The species distribution models demonstrate differing distributions and abundances of host species across the study areas, utilising the strengths of Earth observation data to improve our understanding of landscape and ecological linkages to small mammal distributions and abundances.

## Introduction

Small mammal species form a key role in terrestrial ecosystem functioning in many parts of the world. In addition to their important role in food chains and ecosystem functioning [1–6], understanding their distributions and population dynamics is important for other fields including agriculture [7–10], and public health and disease transmission [11–13]. Many small mammal species exhibit specific habitat preferences which drive the distribution and population dynamics of those species [14–19]. Understanding the linkages between landscape and small mammal ecology is therefore key [11, 20, 21]; when optimal conditions are met, small mammal populations of some species can reach peaks of several hundred individuals per hectare [2, 7, 8, 22]. Therefore, key to understanding the potential impacts of small mammal population dynamics is identifying the distributions and abundances of the species involved [23–25].

Species distribution models (SDM) quantify the environmental conditions leading to species occurrence, and predict potential geographic distributions from existing observations of those species [26], with numerous SDM methods available including machine learning methods such as Random Forests (RF) [27]. SDM encompasses two aspects, explanatory modelling which aims to explain the relationships between a response variable, such as species distribution, and the explanatory variables (e.g., [28]), and predictive modelling, which predicts unknown values of the response variable based on pre-specified relationships [29].

The application of SDMs in this scenario requires suitable small mammal population field data, well spatially distributed across the landscape including the full range of habitats present and differences in species trapability. Field techniques are mostly based on standardized catch effort and transects to collect small mammal indices on regular spaced intervals, depending on the species and habitats studied. Trapline and transect techniques differ in the data type they produce and the spatial scales on which they apply. For example, trapline methods, where multiple traps are set over some hundreds of square metres, can produce measures of species presence and also measures of abundance by combining captures from multiple traps. Transect methods, alternatively, record presence or absence of signs of presence (holes, faeces etc.) at intervals along transect routes, although this can be converted to a continuous occupancy measure by combining multiple intervals [15–17].

While Earth observation (EO) data and derived products have been applied for SDM, for example [30–32], integration of remotely sensed data in SDM remains rare in practice [33]; further opportunities exist to develop SDMs for predictive and explanatory purposes through a close integration of SDM and EO [34]. The broad-scale coverage offered by satellite sensors along with regular revisit periods and cost-free data availability enables characterisation of landscape features and environmental processes underlying species distributions to be quantified and included within SDMs. These include measures of land and vegetation cover [23, 35, 36], structure [37], productivity and phenology [24], forest cover [38] and topographical variables which locally influence biota, habitat structure and growing conditions [39]. EO offers improved monitoring capabilities by filling spatiotemporal data gaps that occur when using field data alone, and predict and monitor short and long-term impacts of management or environmental change [40]. These data products are not yet used to their full potential within SDMs [34]. In particular, vegetation indices (VI) have considerable potential for monitoring vegetation productivity [41], phenology and dynamics [42] which influences the distribution of many small mammal species.

Most SDM studies utilising remote sensing data products use static and temporally aggregated data as predictors [34], with fewer attempts made to utilise time-series data and the dynamic information contained therein [43]. The variation in vegetation state through the growing season is a crucial source of information for discriminating between different types of vegetation [44], with strong potential for quantifying how vegetation biomass change throughout a growing season impacts habitat suitability for different species.

This research presents cost-effective methods integrating in-situ field survey and EO data to identify the important landscape proxy explanatory variables driving small mammal species distributions, and to predictively map spatial patterns of abundance. This research moves from a conventional to a view of “optimal habitat" defined subjectively based on field experience and literature to a view based on a correlation between species distribution and remote sensing variables, whereby the SDM identifies what range of landscape variables influence species abundance. This improves on previous work whereby optimal habitat was defined as simple habitat presence, potentially over-simplifying complex ecological relationships. We follow this by evaluating species-specific predictive abundance maps. This develops a framework for conducting SDM analysis with the flexibility for the method to be applied to different species with varying ecological preferences to identify their optimal habitats.

## Materials and methods

### Study sites

**Fig 1:**
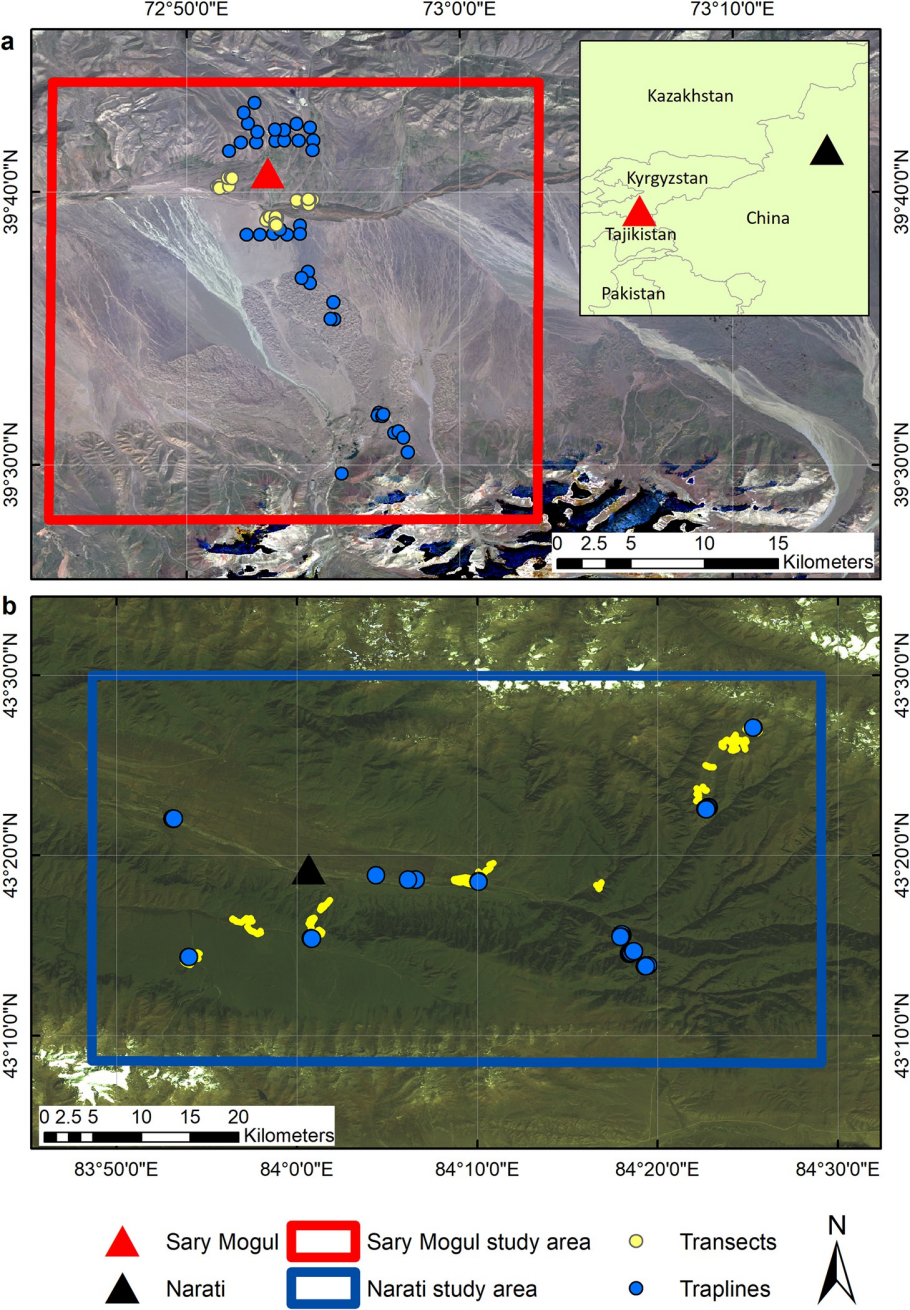
Study site extents and trapline and transect locations. (a) Sary Mogul, Kyrgyzstan, and (b) Narati, China, overlaid on true colour composites of Landsat OLI (Sary Mogul) and Landsat TM (Narati).

This study focussed on two areas, a 55 km x 40 km area around the town of Narati, Yili Valley, Xinjiang, China (43.319°N, 84.016°N), and a 25 km x 30 km area around the village of Sary Mogul, Alay Valley, Kyrgyzstan (39.679°N, 72.883°E) (Fig 1). These sites, whose access is logistically difficult, were selected as they are transmission foci of *Echinococcus multilocularis* (Em), a highly pathogenic parasitic tapeworm for which transmission is linked to small-mammal populations, and consequently were surveyed extensively to establish small mammal species abundance and distributions. Here, analysis focuses specifically on small mammal species distribution modelling at these sites.

**Fig 2:**
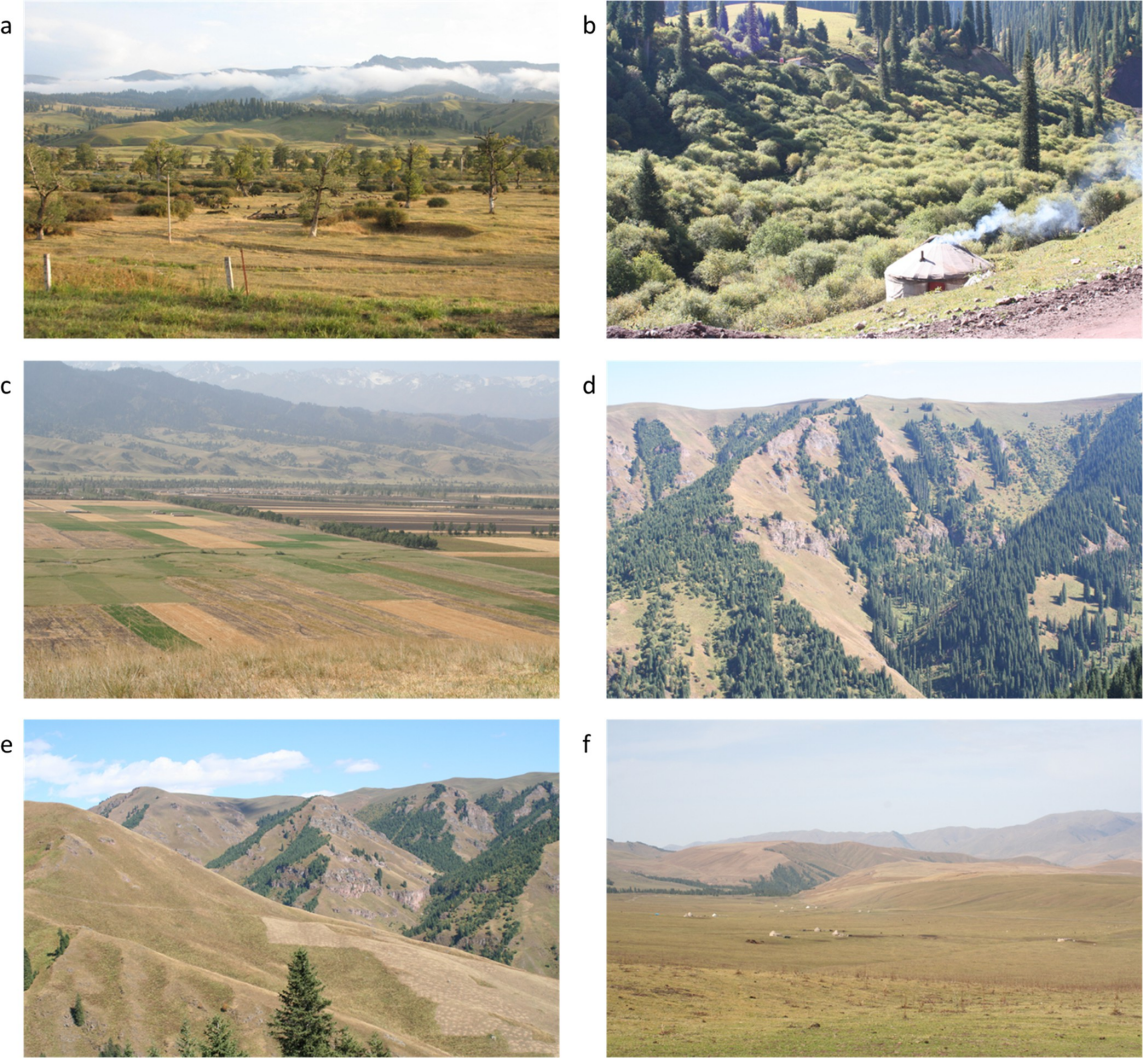
Main habitats present at the Narati study site. (a) Lower-altitude mixed woodland and grassland. (b) Deciduous woodland in valleys. (c) Arable agriculture. (d) Higher-altitude mixed coniferous woodland and grassland. (e) Grassland (including mown areas). (f) High-altitude grassland.

The Narati study site comprises a variety of habitats including river valley, agricultural land, woodland, and semi-natural grassland at altitudes between 1300–3450 m (Fig 2). Higher-altitude areas include heavily grazed grassland and rocky screes. At lower altitudes, longer-grassland areas, in some locations harvested for winter fodder, are present creating a mosaic of longer (uncut) and short (cut) grassland. Areas of coniferous and mixed woodland, often interspersed with grassland patches, are also present, as are extensive areas of seasonal grasslands. Heavy grazing of these grasslands during summer months results in areas becoming bare of vegetation by autumn, especially close to seasonal nomadic settlements. In valley bottoms permanent arable agriculture is present with densely wooded narrow river valleys in places. Scattered settlements are also present, predominantly in river valleys.

**Fig 3:**
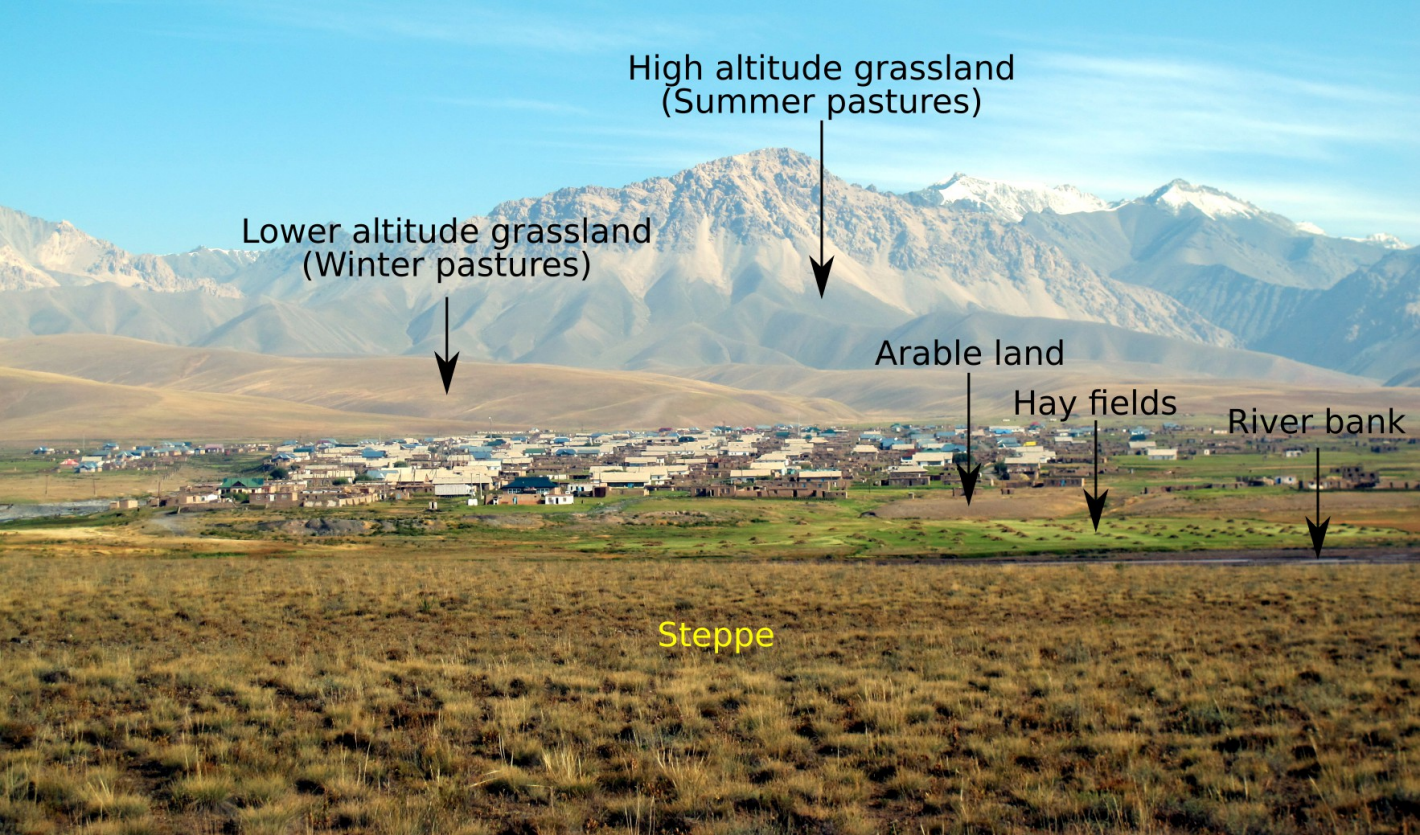
Main habitats present at the Sary Mogul study site.

The Sary Mogul site is located at altitudes between 2900–3200 m on the edge of the Tian Shan and Pamir mountains, and is grassland dominated without woodland (Fig 3). Some areas of low productivity arable areas are present close to built-up areas, along with areas of bushes along river courses and extensive bare areas of dry braided riverbeds. At higher altitudes bare areas are extensively present.

### Small mammal survey

Field surveys were conducted in September 2006 at Narati and September 2014 at Sary Mogul [45] using trapping and transect methods.

#### Trapping

Trapping was performed to establish small mammal distributions but also as part of a larger study to establish Em infection which required specimen autopsy. Small mammals were caught using both small break back traps (sbbt) for animals lighter than 100 g, and big break back traps (bbbt) for larger individuals, with trapping and animal handling carried out in full accordance with the relevant European guidelines (Directive 86/609/EEC) and national regulations. The rodent species investigated in this study do not have protected status, with some even listed as pests and subject to control. The study was carried out as part of several international and national research programmes where protocols have been approved informally by corresponding ethical committees. Similar protocols received also full approval from the Comité d’Ethique Bisontin en Expérimentation Animale (CEBEA No. 58). Each trap was set for three nights (unless non-controlled factors, such as trap theft, dictated otherwise), checked every morning and re-set as necessary. Trapping was undertaken in habitats identified in the field and defined a priori based on dissimilarities in vegetation structure and dominant plant genus composition, with a number of traps grossly proportional to the habitat areas. Standard trapping [15–17] was undertaken in each habitat. Each trapline consisted of 25 traps of a single kind (sbbt or bbbt) spaced 3 m apart, a distance classically selected for providing at least two traps within a small mammal home range. A total of 2910 trap-nights (referring to a single trap set for one night) in 43 traplines were set in Narati, and 3786 trap-nights in 48 traplines in Sary Mogul, with trapped species identified using the references [46–49]. Seven small mammal species were captured at Narati; *Apodemus uralensis* (Pallas, 1811) (Herb field mouse), *Microtus obscurus* (Eversmann, 1841) (Altai vole), *Myodes centralis* (Miller 1906) (Tien Shan red-backed vole), *Sicista tianshanica* (Salensky, 1903) (Tien Shan birch mouse), *Sorex asper* (Thomas, 1914) (Tien Shan shrew), *Ellobius tancrei* (Blasius 1884) (Eastern mole vole) and *Marmota baibacina* (Kastschenko, 1899) (Grey marmot). Three species were captured at Sary Mogul; *Cricetulus migratorius* (Pallas, 1773) (Grey dwarf hamster), *Microtus gregalis* (Pallas, 1779) (Narrow-headed vole) and *E*. *tancrei*. *A*. *uralensis* identifications were confirmed using cytochrome b sequencing and karyotypes. Linnean nomenclature followed [50] except for *M*. *obscurus* which was identified according to [51]. To investigate the influence of trap and control night differences on captures, generalised linear models (GLM) were used with a Poisson link and control night and trap type as explanatory variables. A random effect was added to take into account the fact that controls were made iteratively for each trapline. The logarithm of the total number of traps accessible for a given species (free traps + successful captures after a night) was included as an offset (S1 Table). According to species, the residuals of the model were used as species-specific relative abundance index (termed abundance index below) where trap-type or controls have a statistically significant impact on captures. No spatial autocorrelation was found based on the visual examination of semi-variograms and Moran’s I index, with this analysis (and equivalent analysis for the transect data) performed in R (version 3.6.3) [52].

#### Transects

Transects were used to sample open habitats (grassland, arable fields etc.) for subterranean species, such as *E*. *tancrei*, that cannot be trapped using break-back traps but do leave conspicuous activity indices on the ground surface, and also opportunistically for some trappable species such as *M*. *obscurus*, *M*. *gregalis*, and *M*. *centralis* to provide abundance estimates over a larger range than possible using trapping methods [15–17, 53]. For each transect, 20 intervals of 10 paces were surveyed with activity indicators identifiable to species or genus level (including foraging corridors, ground holes, earth tumuli and small mammal faeces) recorded. Relative abundance scores of small mammal presence (the number of intervals where presence indicators were observed) were produced for each species for each transect. In Sary Mogul, field surveys comprised 37 transects as described in [25]. Transect locations were separated by an average of 1.2 km to avoid spatial autocorrelation [12]. In Narati, 40 similar transects totalling over 41 km were surveyed in grassland areas between 1509–3335 m altitude. Transect routes were selected opportunistically under accessibility constraints in order to cross the largest portion of each habitat patch. They were recorded via Global Positioning System (GPS) receivers with an approximate 15 m accuracy. Abundance indices were computed each 20 intervals of 10 paces to avoid spatial autocorrelation. No evidence of autocorrelation was found based on visual examination of semi-variograms and Moran’s I.

### EO-derived explanatory variables

A suite of EO data products characterising key biophysical factors underlying small mammal distributions including land cover, vegetation temporal variability and topographical variables, were generated. The imagery used to generate these products were coincident with, or acquired as closely as possible to, the field survey years, although persistent cloud cover necessitated a wider image acquisition period at Narati (Table 1). Landsat [54] surface reflectance tier-1 data at 30 m resolution was used, with this data cloud masked to remove pixels affected by cloud, cloud-shadow or snow.

**Table 1:** Number of Landsat images included in the image collections for the respective compositing periods.

Site	Sensor	Acquisition period	Images
Narati	Landsat TM	1^st^ Jan 2006 to 31^st^ Dec 2007	29
Sary Mogul	Landsat OLI	1^st^ Jan 2014 to 31^st^ Dec 2014	87

The imagery collections were used for 1) generating a cloud-free median pixel value composite for land cover classification, and 2) to produce a series of percentile metrics quantifying vegetation index temporal variability across the growing season. Topographical characterisation was performed using 30 m resolution Shuttle Radar Topography Mission (SRTM) Digital Elevation Model (DEM) data, from which slope and aspect were derived.

#### Vegetation temporal variability

Percentile metrics were calculated for a series of vegetation indices derived from the imagery collections for each study area. These included the Normalised Difference Vegetation Index (NDVI), Normalised Difference Water Index (NDWI), Modified Normalised Difference Water Index (MNDWI), Enhanced Vegetation Index (EVI), Green Red Vegetation Index (GRVI), Difference Vegetation Index (DVI), Triangular Vegetation Index (TVI), Spectral Variability Vegetation Index (SVVI), Soil Adjusted Vegetation Index (SAVI), and Tasselled Cap brightness, greenness and wetness (see S2 Table for details). Percentile metrics were calculated for each VI for the 5th, 10th, 25th, 50th, 75th, 90th and 95th percentiles across the imagery acquisition period, with VI range and range of 75th-25th, 90th-10th and 95th-5th percentiles additionally calculated. Percentiles were used as they capture the dynamics of the phenological response of the vegetation, but detach it from the specific timing of the event [55]. This is important as phenological events move slightly from year to year in response to climate.

#### Land cover classification

Image classification was used to create the required land cover classifications. The image classification process required an input data stack, which comprised of satellite data (Table 1) and contextual data (topographical data), plus training areas for each of the land cover classes. The image data stack used multi-temporal composite data created from one year of Landsat data (Table 1), as this enabled the production of cloud-free images, which are known to perform well in image classifications [56]. Specifically, the image data stack comprised of: median composites of the Landsat bands (all bands except the thermal?), NDVI vegetation temporal variability (10th and 90th percentile metrics and the 10th to 90th percentile range), and topographical bands (elevation, slope and aspect).

The land cover classification was based on eight-classes comprising grassland, woodland, arable, bushes, built-up, bare, water and snow (Figs 2 and 3 illustrate the land cover classes present). Woodland was absent from Sary Mogul.

Reference locations of known land cover types were used for classification training and accuracy assessment respectively, and were collected from: 1) field locations of known land cover class (recorded via GPS); 2) reference locations derived from field photographs; 3) visual interpretation of VHR satellite imagery available via Google Earth, and; 4) expert knowledge of clear imagery features (e.g. water and snow). Using higher-resolution imagery as reference data is an established technique [57], with Google Earth previously used for this purpose [58]. Reference locations were allocated on an alternating basis for training or validation, creating for Sary Mogul 352 training and validation locations each (704 in total). For Narati, 800 validation points (100 per class) were used. Where land cover homogeneity allowed training locations were used to generate larger training polygons.

Once the training areas and the data stack had been created, a 200-tree random forest [59] image classification was run. To train the classifier, for each class, 5000 training pixels were selected from within the class-specific training polygons using random stratified sampling. This process was applied to the Narati and Sary Mogul study areas separately creating two final land cover classifications (see Results section for outputs and validation).

The land cover classifications were then used to derive the proportional presence of grassland, woodland and arable across the study areas, using moving windows with nested kernel sizes of 50–500 m, in 50 m increments. Data for each EO metric was extracted for each trapline and transect interval location. This data was then used in the predictive species modelling. All EO data processing, including the Random Forest classification, was performed using Google Earth Engine (GEE) [60].

### Predictive species distribution modelling

To determine which EO variables were important in relation to small mammal abundance, the boruta feature selection approach was used to identify and retain only those variables statistically important for each species [61]. This was performed in R (v4.0.3) using the Boruta (v7.0.0; [61]) and randomForest (v4.6.14, [62]) packages. Random Forests (RF), were then applied in a regression (rather than classification) capacity to assess their effectiveness for predictively modelling abundances, using these reduced sets of important variables, for each species, at each site. RF hyperparameter tuning was performed, with the internal RF parameters (number of trees, minimum leaf population, maximum nodes, number of variables per split and bag fraction) tuned iteratively to identify the best performing model, determined by the highest coefficient of determination (R^2^) value when comparing predicted values to the observed values for the validation data set. With the optimal RF parameters established, each species-specific model was applied predictively for both study areas. Model validation was performed using leave-one-out cross validation, producing R^2^ values of observed versus predicted values.

The areas of optimal habitat were then calculated for each species based on the combination of EO variables included in each SDM. The ratio of optimal habitat to total land evaluates the risk for a species to develop outbreaks and reach large population densities, within the limits of its ecology and the biotic capacity of the habitat. To convert the SDM continuous measure of predicted abundance to binary optimal / non-optimal classes, a thresholding approach was applied whereby the mean predicted abundance of all data used to build the SDM was set as the threshold value [63]. Predicted abundance values above this threshold value were classified to optimal, values below were non-optimal. This approach has been used previously for the maximum entropy SDM method where the predicted probability of presence values generated are converted to binary presence-absence values. [64] determined that this average probability approach is at least as good as more complex approaches to determining threshold values.

## Results

### Land cover mapping

**Fig 4:**
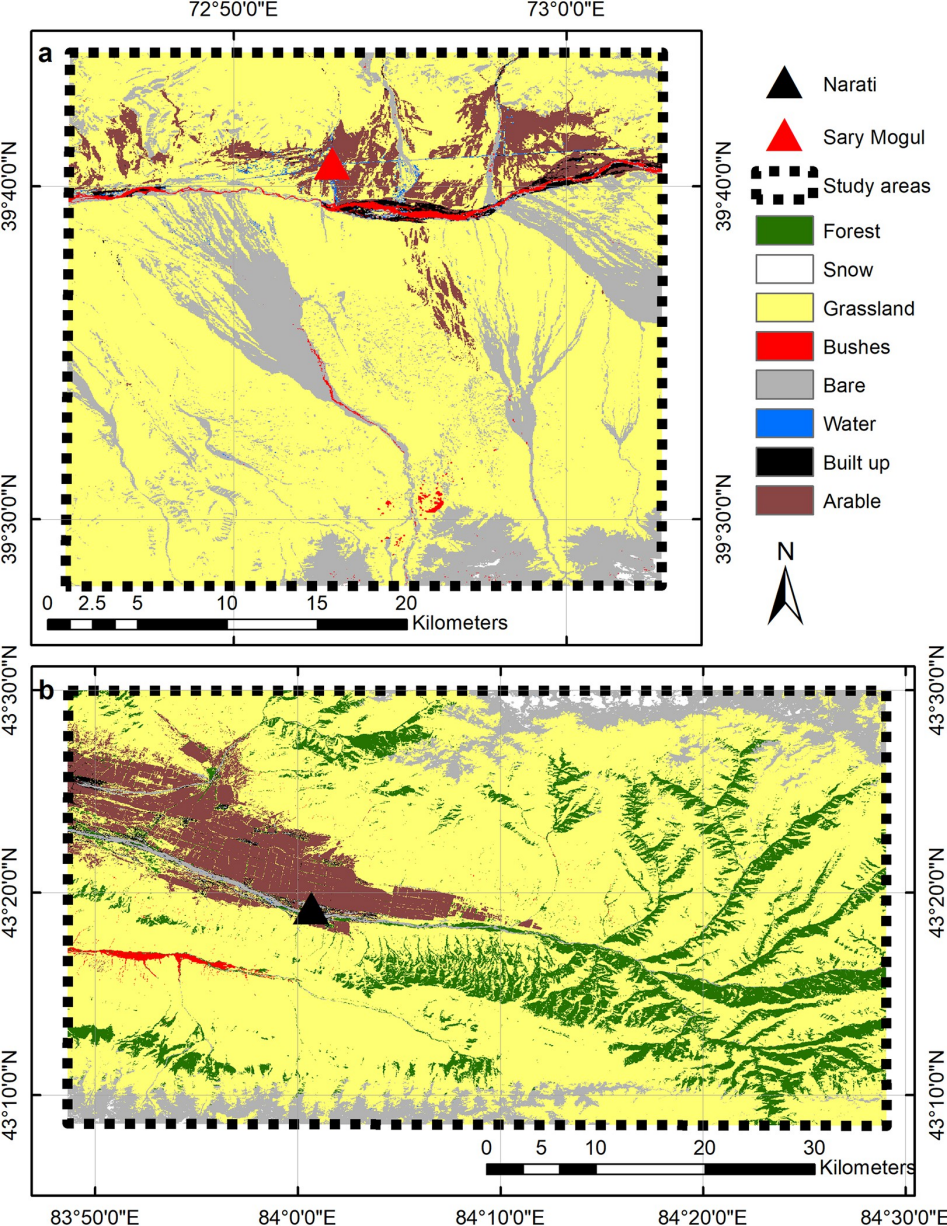
Land cover classifications. (a) Sary Mogul study area. (b) Narati study area.

Land cover classifications were generated for both study areas (Fig 4), with Sary Mogul comprising 72.16% grassland, 20.80% bare, 4.76% arable, 0.77% bushes, 0.57% snow, 0.55% built-up, and 0.39% water. Narati comprised 68.54% grassland, 12.93% forest, 10.37% bare, 6.57% arable, 0.71% snow, 0.55% built-up, 0.32% bushes, and 0.01% water. Classification accuracies for Sary Mogul and Narati are 85.23% and 94.50% respectively, with confusion matrices presented in S3 and S4 Tables.

### Boruta feature selection

The species-specific variable sets identified as important by the boruta feature selection were then used as the explanatory variables for the SDM. The top five variables for each species are presented in Tables 2 and 3, with complete lists in S5–S7 Tables.

**Table 2:** Top five EO-derived variables identified by the boruta feature selection analysis as important for each small mammal species for Narati.

Trapline					Transect	
*A*. *uralensis*	*M*. *obscurus*	*M*. *centralis*	*S*. *tianshanica*	*S*. *asper*	*E*. *tancrei*	*M*. *baibacina*
TCB 10p range	MNDWI 25p range	SVVI 50p	SAVI 10p	TCW 50p	NDVI 50p	Grassland 400m
Woodland 450m	TCB 10p range	EVI 50p	Elevation	NDWI 90p	Elevation	Woodland 500m
Woodland 400m	GRVI 90p	TCW 50p	NDVI 10p	Woodland 100m	EVI 50p	TCB 10p
Woodland 500m	NDWI 10p	EVI 10p range	NDWI 10p range	TCW 25p	TCG 50p	TCB 25p
Elevation	Grassland 250m	MNDWI 25p range	SAVI 10p range	Woodland 150m	NDWI 90p range	TCB 50p

TCB = Tasseled Cap Brightness, TCW = Tasseled Cap Wetness, TCG = Tasseled Cap Greeness, EVI = Enhanced Vegetation Index, NDWI = Normalised Difference Water Index, NDVI = Normalised Difference Vegetation Index, GRVI = Green Red Vegetation Index, MNDWI = Modified Normalised Difference Water Index, SVVI = Spectral Variability Vegetation Index, SAVI = Soil Adjusted Vegetation Index, 10p = 10th percentile, 25p = 25th percentile, 50p = 50th percentile, 90p = 90th percentile. Variables are displayed in order of decreasing importance as determined by the random forest variable importance rankings, with the most important variable at the top (see S5 and S6 Tables for full tables).

**Table 3:** Top five EO-derived variables identified by the boruta feature selection analysis as important for each small mammal species for Sary Mogul.

Trapline		Transect	
*M*. *gregalis*	*C*. *migratorius*	*E*. *tancrei*	*M*. *gregalis*
TCB 25P	NDWI 25p range	TCW 10p	TVI 50p
TCB 50P	NDWI 10P	DVI 25p range	DVI 50p
Grassland 250m	TVI 75P	TCG 25p range	NDWI 50p
Grassland 300m	GRVI 25p range	SAVI 25p range	
Grassland 500m	NDWI 75P	NDVI 25p range	

TCB = Tasseled Cap Brightness, TCW = Tasseled Cap Wetness, TCG = Tasseled Cap Greeness, NDWI = Normalised Difference Water Index, NDVI = Normalised Difference Vegetation Index, TVI = Triangular Vegetation Index, GRVI = Green Red Vegetation Index, DVI = Difference Vegetation Index, SAVI = Soil Adjusted Vegetation Index, 10p = 10th percentile, 25p = 25th percentile, 50p = 50th percentile, 75p = 75th percentile. Variables are displayed in order of decreasing importance as determined by the random forest variable importance rankings, with most important variable at the top (see S7 Table for full table).

The boruta results (Tables 2 and 3) demonstrated that the most important EO-derived variables for each species varied, with proportional presence of woodland being important for *A*. *uralensis*, *S*. *asper* and *M*. *baibacina* at Narati, with grassland being important for *M*. *obscurus* and *M*. *baibacina* at Narati, and *M*. *gregalis* at Sary Mogul. Both vegetation and water indices were consistently identified as important, although the specific index and percentile/range value did vary between species. Elevation is also identified as being amongst the top five most important variables for *A*. *uralensis*, *S*. *tianshanica* and *E*. *tancrei* at Narati.

For all sites, trapping methods and small mammal species, the RF hyperparameter tuning determined the RF model parameters producing the highest R^2^ values between predicted and observed values as: number of trees = 200, minimum leaf population = 1, maximum nodes = unlimited, and variables per split = √number of variables, bag fraction = 0.7. RF parameter tuning results are available in S8–S10 Tables.

### Species distribution modelling

#### Narati

**Fig 5:**
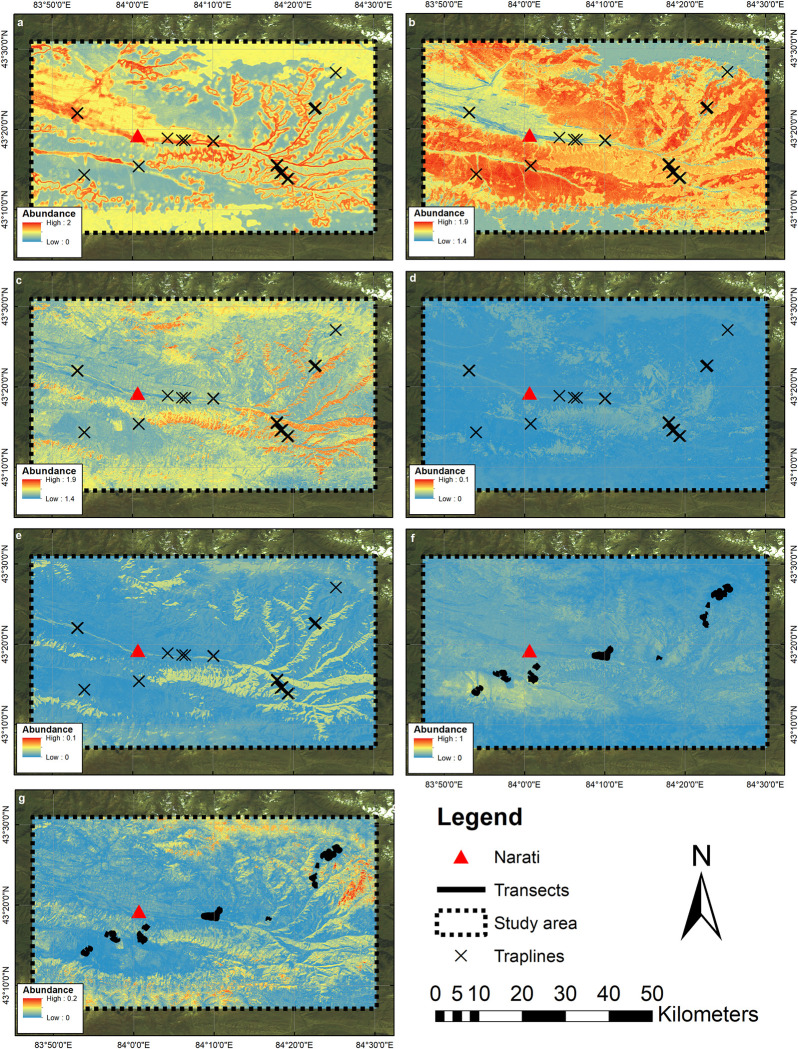
Random forest predicted abundance for the Narati study area. (a) *A*. *uralensis*, (b) *M*. *obscurus*, (c) *M*. *centralis*, (d) *S*. *tianshanica* and (e) *S*. *asper* using trapline data, and (f) *E*. *tancrei*, and (g) *M*. *baibacina* using transect data.

RF analysis indicated variability in predicted abundance patterns across the Narati study area (Fig 5).

Results from SDM analysis of trapline data indicated highest predicted abundances to be located in woodland-grassland boundary areas and wetter areas close to watercourses for *A*. *uralensis*, in drier, higher-biomass grassland dominated areas for *M*. *obscurus*, and in wetter areas with high levels of woodland cover for *M*. *centralis*. Although predicted trapping success for *S*. *tianshanica* and *S*. *asper* were considerably lower, where higher trapping success were predicted for *S*. *tianshanica* this was at lower elevations in higher biomass areas comprising grassland, woodland and bushy areas along watercourses. For *S*. *asper*, higher abundances were predicted predominantly in areas of high woodland cover and with increasing wetness close to watercourses. SDM of the transect data indicated highest predicted abundances for *E*. *tancrei* in grassland dominated areas. Areas of higher *M*. *baibacina* abundance were predicted in higher-altitude grasslands, particularly on slopes. For the transect data, higher predicted abundance of *E*. *tancrei* corresponded to grassland dominated areas, while for *M*. *baibacina* this corresponded to grasslands at higher elevation and topographically variable (sloped) areas.

#### Sary Mogul

**Fig 6:**
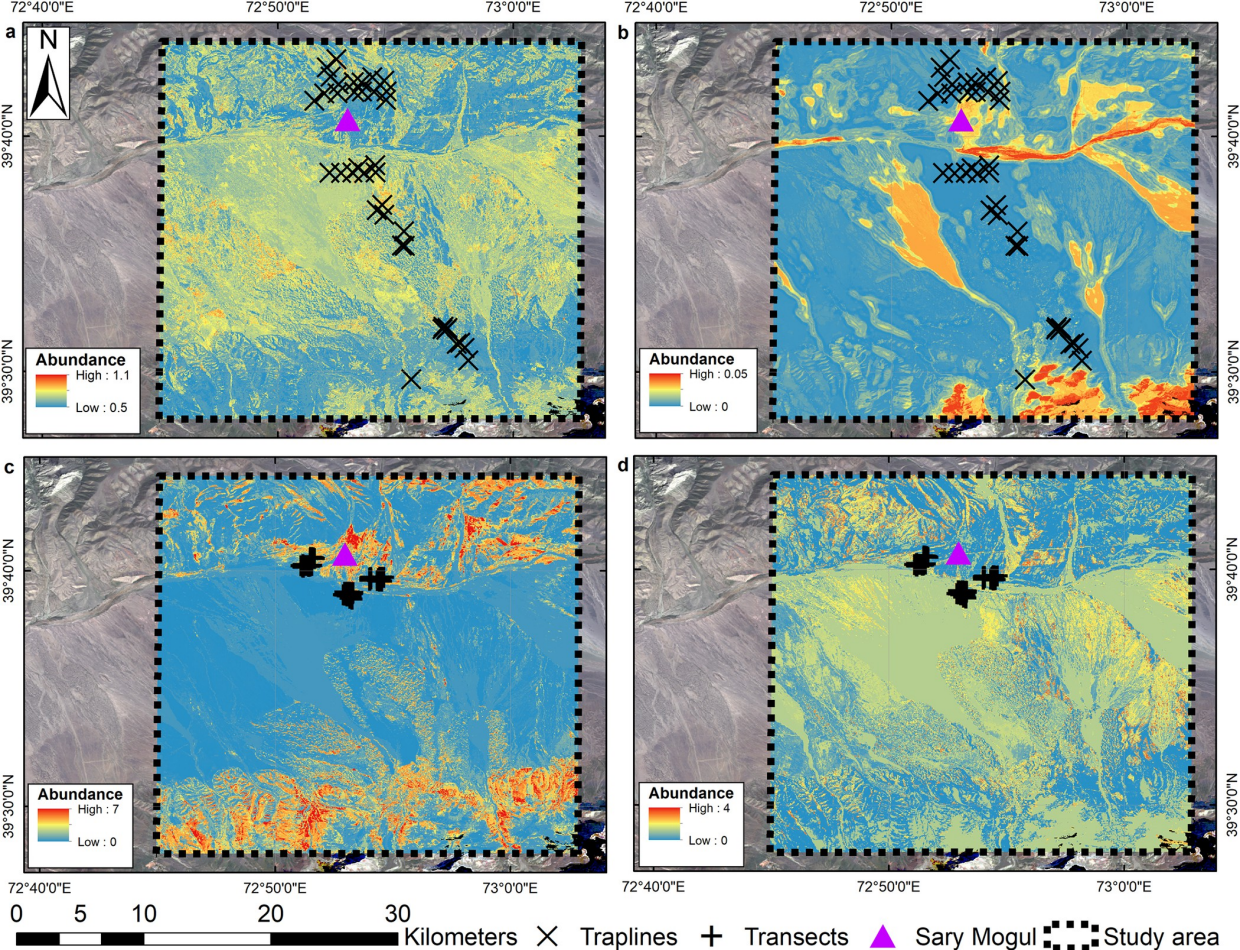
Random forest predicted abundance for the Sary Mogul study area. (a) *C*. *migratorius* and (b) *M*. *gregalis* using the trapline survey data, and (c) *E*. *tancrei* and (d) *M*. *gregalis* using the transect survey data.

For Sary Mogul, trapline SDMs predicted generally low abundance of *C*. *migratorius* (Fig 6). Where higher abundances were predicted, these corresponded with sparsely-vegetated areas where the annual VI range was lower, indicating areas with consistently low biomass levels across the growing season are preferred. Contrastingly, lower abundances are observed in arable areas. For *M*. *gregalis*, generally low abundances were predicted, although higher abundances were predicted in shrub areas close to watercourses and arable areas, sparsely vegetated dry riverbed areas, and higher elevation grasslands. Conversely, very low predicted abundances were observed for broader expanses of sparsely vegetated areas.

Transect data analysis for *E*. *tancrei* predicted extensive low abundance in sparsely vegetated areas, with higher abundance predicted in arable areas, more productive vegetated areas along watercourses and where higher wetness levels are maintained at drier times of the year, and higher elevation grasslands. For *M*. *gregalis* lower predicted abundances corresponded with some grassland and arable areas, although there is considerable local variability with localised hotspots of higher abundance predicted in grassland, arable and sparsely vegetated areas.

#### Leave-one-out cross validation

The leave-one-out cross validation results indicated good performance of the SDMs (Table 4), with R^2^ values ranging from 0.670 for *M*. *gregalis* transect data from Sary Mogul to 0.939 for *E*. *tancrei* transect data from Narati. R^2^ values were broadly similar for trapline and transect methods, although there is variability between species.

**Table 4:** Leave-one-out R^2^ values for the random forest species distribution models.

Site	Method	Species	R^2^
Narati	Trapline	*A*. *uralensis*	0.780
		*M*. *obscurus*	0.907
		*M*. *centralis*	0.886
		*S*. *tianshanica*	0.771
		*S*. *asper*	0.835
	Transect	*E*. *tancrei*	0.939
		*M*. *baibacina*	0.878
Sary Mogul	Trapline	*C*. *migratorius*	0.830
		*M*. *gregalis*	0.775
	Transect	*E*. *tancrei*	0.810
		*M*. *gregalis*	0.670

#### Percentage of optimal habitat in the total area

Here, areas of optimal habitat were computed for each species based on the thresholding values and combination of EO variables included in each SDM (Table 5). This showed considerable variability in the area of optimal habitat for the different species at each study area, at Narati varying from 16.8% of the total study area comprising optimal habitat for *S*. *asper*, to 75.1% for *M*. *baibacina*, and at Sary Mogul from 30.6% for *E*. *tancrei* to 53.5% for the *M*. *gregalis* transect data.

**Table 5:** Proportion of optimal habitats in the total land, based on species-specific predictive models, and model-specific threshold values applied.

Site	Method	Species	Threshold value	Optimal habitat area (km^2^)	% Total land
Narati	Trapline	*A*. *uralensis*	1.136	504.5	21.4
		*M*. *obscurus*	1.740	1357.6	57.5
		*M*. *centralis*	1.599	573.0	24.3
		*S*. *tianshanica*	0.006	664.0	28.2
		*S*. *asper*	0.014	395.8	16.8
	Transect	*E*. *tancrei*	0.078	1162.7	49.4
		*M*. *baibacina*	0.009	1768.9	75.1
Sary Mogul	Trapline	*C*. *migratorius*	0.665	313.2	41.3
		*M*. *gregalis*	0.007	247.7	32.7
	Transect	*E*. *tancrei*	0.928	231.7	30.6
		*M*. *gregalis*	1.047	405.6	53.5

## Discussion

This study assessed the effectiveness of RF SDM for predictively modelling abundance for nine small mammal species. The objective, to identify important landscape proxy variables driving small mammal distributions and generate species-specific predictive abundance maps has been achieved. This is evidenced by the high leave-one-out cross validation R^2^ values for the SDM models, ranging from 0.670 for the *M*. *gregalis* transect data at Sary Mogul to 0.939 for the *E*. *tancrei* transect data at Narati, demonstrating the majority of variance to be explained by the EO variables. These EO variables characterised the landscape in terms of land cover distributions, topographical variability and vegetation and wetness dynamics across the growing season via the VI and water index (WI) percentile products. This enabled examination of the impact of low, mid and high vegetation and moisture proxy variables on the small mammal species in question.

SDM predicted high abundance areas varied considerably for each species; at Narati grasslands were predicted to hold higher abundances of *M*. *obscurus*, *E*. *tancrei* and *M*. *baibacina*, forest areas hold higher abundances of *M*. *centralis* and *S*. *asper*, with mixed forest—grassland boundary areas and areas close to watercourses predicted to hold higher abundances of *A*. *uralensis* and *S*. *tianshanica*. However, it is not simply predominant land cover type influencing species abundance, but also further variables characterising localised variability in vegetation and wetness condition. For example, whereas grassland is identified as the key land cover type in relation to abundance of *M*. *obscurus*, a range of VI and WI variables are also important in the SDM, demonstrating increasing abundances with higher VI values and decreasing abundances with higher WI values, indicating preferences for higher biomass, drier grassland areas. Similarly, for other species, the variables ranked as important comprised a mix of land cover and vegetation and water index metric variables.

This advances the findings of previous studies, for example [7, 8, 21], that have modelled small mammal distributions based on only the ratio of optimal to marginal patch area (ROMPA) for a specific species within the broader landscape [65], using a pre-defined decision of what is considered key habitat type for a species. Characterising ROMPA simply through the extent of a discrete land cover type across an area of interest precludes examination of how variability within land cover classes drives small mammal distributions and abundances, and so offers a restricted understanding of the mechanics driving those patterns. The approach used here overcomes this, and at least objectively identifies good proxies to optimal habitat for a species based not just on the proportional coverage of discrete land cover type(s), but additionally on the vegetation and wetness conditions, and temporal dynamics thereof, of a given area. This differs from the conventional ROMPA approach, as here each species informs us through a specific predictive model which variables (combination of EO variables) form its own optimal habitat. For instance, where grassland was estimated at approximately 70% of total land both in Sary Mogul and Narati, grassland optimal habitat for *E*. *tancrei* was predicted to be only around 30 and 50% respectively. Hence here, we move from a human perspective to a species perspective of optimal habitat.

When evaluating these results, it is necessary to again consider small mammal ecology. For instance, Eulipotyphla insectivores (e.g. *S*. *asper*) cannot reach high densities since they are situated at a high level in the trophic chain. *M*. *centralis* is a forest vole, and generally forest species do not reach as high population densities over large areas as grassland voles do [14, 66, 67]. Conversely, *E*. *tancrei* and *M*. *obscurus* are grassland voles, and in areas of high availability of optimal habitat their populations can reach very high densities over large areas [7–8]. Whereas alternative SDM methods such as Maximum Entropy can predict species presence [68], it is the ability of random forests to identify high abundance peaks of small mammals, rather than necessarily just their presence, that is of particular value in determining their function within an ecosystem. It must be acknowledged, however, that a limitation is that interannual variations in small mammal populations cannot be captured via trapping/transects from a single snapshot in time. Consequently, this means some areas exhibiting low abundance or virtual absence of a species at the time of the study could potentially be at high abundance some months/years later and conversely [66] at different stages of their population cycle.

The potential of EO data to characterise a wider range of biophysical environmental variables for SDMs has been strongly suggested here. EO datasets can contribute to future monitoring programmes, complementing field observations by offering broader spatial and temporal coverage. As such, synergies between EO, ecological modelling communities and field ecologists will yield considerable benefits in improving modelling and predictions of species distributions over broad scales, including filling data gaps, improved characterisation of environmental variables influencing species distributions, and through effective, repeatable and cost-effective monitoring of ecological systems [34]. As extensive historical remote sensing data archives exist, including up to five decades of historical data for Landsat, there is also potential for quantifying population responses to landscape change [69] including lag times between landscape modification and subsequent population change. The inclusion of temporally aggregated VI variables characterising vegetation temporal variability throughout the growing season, rather than just vegetation condition from a single snap-shot in time, also overcomes previous limitations and enables inclusion of vegetation dynamic variables within SDMs. However, the use of EO-data in SDM’s (and the development of SDM’s generally) is very dependent on the availability of suitable ground data. Specifically, good quality ground data collected from a spatially representative set of sites. Here, we retrospectively applied SDM’s to existing data, but future work at these sites could build on this work.

These methods will continue to leverage the strengths of EO data to improve our understanding of landscape and ecological linkages to small mammal distributions and population dynamics.

## Supporting information

S1 Table Selection of small mammal abundance indices according to the effects detected based on Poisson GLM. (DOCX)

S2 Table Details of the vegetation indices calculated from the Sentinel-2 and Landsat data. (DOCX)

S3 Table Confusion matrix for the Sary Mogul land cover classification. (DOCX)

S4 Table Confusion matrix for the Narati land cover classification. (DOCX)

S5 Table Remote sensing variables identified by the boruta feature selection analysis as important for each small mammal species for the Narati trapline data. (DOCX)

S6 Table Remote sensing variables identified by the boruta feature selection analysis as important for each small mammal species for the Narati transect data. (DOCX)

S7 Table Remote sensing variables identified by the boruta feature selection analysis as important for each small mammal species for Sary Mogul. (DOCX)

S8 Table Random forest hyperparameter tuning results for the Narati trapline data, displaying R^2^ values between predicted and observed values using leave-one-out cross validation. n = number of trees, MLP = minimum leaf population, MN = maximum nodes, VPS = variables per split, BF = bag fraction.
(DOCX)

S9 Table Random forest hyperparameter tuning results for the Narati transect data, displaying R^2^ values between predicted and observed values using leave-one-out cross validation. n = number of trees, MLP = minimum leaf population, MN = maximum nodes, VPS = variables per split, BF = bag fraction.
(DOCX)

S10 Table Random forest hyperparameter tuning results for Sary Mogul, displaying R^2^ values between predicted and observed values using leave-one-out cross validation. n = number of trees, MLP = minimum leaf population, MN = maximum nodes, VPS = variables per split, BF = bag fraction.
(DOCX)
