# Supplementary material for: Mapping small mammal optimal habitats using satellite-derived proxy variables and species distribution models
Source: PLoS One. 2023 Aug 17;18(8):e0289209. doi: 10.1371/journal.pone.0289209 (PMC10434852; doi:10.1371/journal.pone.0289209)
Supplement: S1 Table — (DOCX) [file pone.0289209.s001.docx]

# Supplementary information

To investigate the influence of trap and control night differences on captures, generalized linear models (GLM) with a Poisson link were used (Table S1). The response variable was the number of captures of a given species, and the independent variables the control night (1,2,3) and the trap type; an offset was added as the logarithm of the total number of traps minus those having caught another species. For all species in Sary Mogul and for *Sicista tianshanica* in Narati, no statistical differences were detected between trap nights and trap types, therefore the abundance index was defined as $a_{tsp}=\frac{n_{tsp}}{(c_{t}-n_{osp})}$, with n_tsp_ the number of captures of the target species divided by the sum ‘total number of control nights – number of captures of other species + the number of capture of the target species’, c_t_ the total number of control nights and n_osp_ the total number of capture of other species (once a trap is triggered it cannot therefore catch the target species until it is reset). For the other species for which statistical differences were found either between trap types or/and control nights, the abundance index was defined as the residuals (shifted to zero at the minimum value) of the Poisson GLM modelling those differences.

**S1 Table. Selection of small mammal abundance indices according to the effects detected based on Poisson GLM.**

| Species | Trap type | Control night | Study area | Abundance index |
| --- | --- | --- | --- | --- |
| *Apodemus uralensis* | X |  | Narati | residuals |
| *Cricetulus migratorius* |  |  | Sary Mogul | a_tsp_ |
| *Microtus gregalis* |  |  | Sary Mogul | a_tsp_ |
| *Microtus obscurus* | X | X | Narati | residuals |
| *Myodes centralis* |  | X | Narati | residuals |
| *Sicista tianshanica* |  |  | Narati | a_tsp_ |
| *Sorex asper* | X |  | Narati | a_tsp_* |

X, model coefficient significantly different from zero; * excluding BBBT (BBBT are too large to trap this species).
