# Supplementary material for: Mapping small mammal optimal habitats using satellite-derived proxy variables and species distribution models
Source: PLoS One. 2023 Aug 17;18(8):e0289209. doi: 10.1371/journal.pone.0289209 (PMC10434852; doi:10.1371/journal.pone.0289209)
Supplement: S2 Table — (DOCX) [file pone.0289209.s002.docx]

**S2 Table. Details of the vegetation indices calculated from the Sentinel-2 and Landsat data.**

| **Index** | **Equation** | **Reference** |
| --- | --- | --- |
| **NDVI** – Normalised Difference Vegetation Index | $\frac{(NIR-red)}{(NIR+red)}$ | [1] |
| **NDWI** - Normalised Difference Water Index | $\frac{(NIR-SWIR1)}{(NIR+SWIR1)}$ | [2,3] |
| **MNDWI** - Modified Normalised Difference Water Index | $\frac{(Green-SWIR)}{(Green+SWIR)}$ | [3] |
| **EVI** – Enhanced Vegetation Index | $2.5*\frac{(NIR-red)}{(NIR+6 *red-7.5*blue+1)}$ | [4] |
| **GRVI** – Green red vegetation index | $\frac{(red-green)}{(red+green)}$ | [5] |
| **DVI** – Difference vegetation index | $NIR-red$ | [6] |
| **TVI -** Triangular Vegetation Index | $0.5\left( 120\left( NIR-green \right)-200\left( red-green \right) \right)$ | [7] |
| **SVVI** - Spectral Variability Vegetation Index | $SD\left( blue, green, red, NIR, SWIR1, SWIR2 \right)-SD(NIR, SWIR1, SWIR2)$ | [8] |
| **Tasseled cap**  Brightness  Greenness  Wetness | $b_{1}blue+b_{2}green+b_{3}red+b_{4}NIR+b_{5}SWIR1+b_{6}SWIR2$  $g_{1}blue+g_{2}green+g_{3}red+g_{4}NIR+g_{5}SWIR1+g_{6}SWIR2$  $w_{1}blue+w_{2}green+w_{3}red+w_{4}NIR+w_{5}SWIR1+w_{6}SWIR2$ | [9] |
| **SAVI** – Soil Adjusted Vegetation Index | $\frac{(1+L)(NIR-Red)}{(NIR+Red+L)}$ | [10] |

Table S2 provides the Tasselled cap coefficients for Landsat. SD is the standard deviation. NIR is the Near Infrared, SWIR is the Short-Wave Infrared.

**References**

1. Rouse JW, Haas RH, Deering DW, Schell JA, Harlan JC. Monitoring the vernal advancement and retrogradation (green wave effect) of natural vegetation; E73-10693; NASA: Greenbelt, MD, USA, 1973**;** p. 112.
2. McFeeters SK. The use of Normalized Difference Water Index (NDWI) in the delineation of open water features. Int. J. Remote Sens. 1996; 17(7): 1425–1432.
3. Xu H. Modification of normalised difference water index (NDWI) to enhance open water features in remotely sensed imagery, Int. J. Remote Sens*.* 2006; 27(14): 3025-3033.
4. Liu HQ, Huete A. A feedback based modification of the NDVI to minimize canopy background and atmospheric noise. IEEE Trans. Geosci. Remote. Sens**.** 1995; 33(2): 457–465.
5. Gitelson AA, Kaufman YJ, Merzlyak M. Use of a green channel in remote sensing of global vegetation from EOS- MODIS. Remote Sens. Environ. 1996; 58(3): 289-298.
6. Jordan CF. Derivation of Leaf-Area Index from Quality of Light on the Forest Floor, Ecology. 1969; 50(4): 663-666.
7. Broge NH, Leblanc E. Comparing prediction power and stability of broadband and hyperspectral vegetation indices for estimation of green leaf area index and canopy chlorophyll density. Remote Sens. Environ*.* 2001; 76(2): 156-172.
8. Coulter LL, Stow DA, Tsai YH, Ibanez N, Shih HC, Kerr A, et al. Classification and assessment of land cover and land use change in southern Ghana using dense stacks of Landsat 7 ETM+ imagery. Remote Sens. Environ*.* 2016; 184: 396–409.
9. Crist EA. Tasseled Cap Equivalent Transformation for Reflectance Factor Data. Remote Sens. Environ*.* 1985; 17(3): 301–306.
10. Huete AR. A Soil Adjusted Vegetation Index (SAVI), Remote Sens. Environ*.* 1998; 25(3): 295-309.
