# Supplementary material for: Mapping small mammal optimal habitats using satellite-derived proxy variables and species distribution models
Source: PLoS One. 2023 Aug 17;18(8):e0289209. doi: 10.1371/journal.pone.0289209 (PMC10434852; doi:10.1371/journal.pone.0289209)
Supplement: S3 Table — (DOCX) [file pone.0289209.s003.docx]

**S3 Table. Confusion matrix for the Sary Mogul land cover classification.**

| Classified data | Reference data | | | | | | |
| --- | --- | --- | --- | --- | --- | --- | --- |
|  | Grassland | Water | Bare | Built-up | Bushes | Agriculture | Users accuracy (%) |
| Grassland | 141 | 0 | 4 | 0 | 0 | 5 | 94.00 |
| Water | 0 | 33 | 5 | 0 | 0 | 0 | 86.84 |
| Bare | 0 | 1 | 48 | 0 | 0 | 1 | 96.00 |
| Built-up | 6 | 0 | 8 | 7 | 0 | 4 | 28.00 |
| Bushes | 2 | 0 | 2 | 2 | 30 | 3 | 76.92 |
| Agriculture | 9 | 0 | 0 | 0 | 0 | 41 | 82.00 |
| Producers accuracy (%) | 89.24 | 97.06 | 71.64 | 77.78 | 100.00 | 75.93 |  |
|  | Overall accuracy (%) = 85.23 | | | | | | |
