# Supplementary material for: Mapping small mammal optimal habitats using satellite-derived proxy variables and species distribution models
Source: PLoS One. 2023 Aug 17;18(8):e0289209. doi: 10.1371/journal.pone.0289209 (PMC10434852; doi:10.1371/journal.pone.0289209)
Supplement: S4 Table — (DOCX) [file pone.0289209.s004.docx]

**S4 Table. Confusion matrix for the Narati land cover classification.**

| Classified Data | Reference Data | | | | | | | | |
| --- | --- | --- | --- | --- | --- | --- | --- | --- | --- |
|  | Snow | Water | Bare | Built up | Bushes | Arable | Grassland | Forest | Users accuracy (%) |
| Snow | 100 |  |  |  |  |  |  |  | 100.00 |
| Water |  | 100 |  |  |  |  |  |  | 100.00 |
| Bare |  |  | 100 |  |  |  |  |  | 100.00 |
| Built up |  | 3 | 3 | 65 |  | 29 |  |  | 65.00 |
| Bushes |  |  |  |  | 95 |  | 2 | 3 | 95.00 |
| Arable |  |  |  |  |  | 100 |  |  | 100.00 |
| Grassland |  |  |  |  |  |  | 100 |  | 100.00 |
| Forest |  |  |  |  |  |  | 4 | 96 | 96.00 |
| Producers accuracy (%) | 100.00 | 97.09 | 97.09 | 100.00 | 100.00 | 77.52 | 94.34 | 96.97 |  |
|  | Overall accuracy (%) = 94.50 | | | | | | | | |
