# Supplementary material for: Mapping small mammal optimal habitats using satellite-derived proxy variables and species distribution models
Source: PLoS One. 2023 Aug 17;18(8):e0289209. doi: 10.1371/journal.pone.0289209 (PMC10434852; doi:10.1371/journal.pone.0289209)
Supplement: S5 Table — (DOCX) [file pone.0289209.s005.docx]

**S5 Table. Remote sensing variables identified by the boruta feature selection analysis as important for each small mammal species for the Narati trapline data.**

| **Trapline** |  |  |  |  |
| --- | --- | --- | --- | --- |
| *A. uralensis* | *M. obscurus* | *M. centralis* | *S. tianshanica* | *S. asper* |
| TCB 10p range | MNDWI 25p range | SVVI 50p | SAVI 10p | TCW 50p |
| Woodland 450m | TCB 10p range | EVI 50p | Elevation | NDWI 90p |
| Woodland 400m | GRVI 90p | TCW 50p | NDVI 10p | Woodland 100m |
| Woodland 500m | NDWI 10p | EVI 10p range | NDWI 10p range | TCW 25p |
| Elevation | Grassland 250m | MNDWI 25p range | SAVI 10p range | Woodland 150m |
| Grassland 400m | NDVI 75p | DVI 10p | NDWI 90p | Woodland 200m |
| Woodland 300m | Elevation | TCW 75p | NDVI 10p range | GRVI 10p |
| Grassland 450m | Grassland 300m | TVI 10p | MNDWI 5p | Woodland 50m |
| Grassland 500m | NDWI 5p | EVI 5p |  | TVI 10p |
| Woodland 350m | NDVI 95p | EVI 90p |  | TCW 75p |
| Grassland 350m | Grassland 200m | TCB 10p |  | MNDWI 75p |
| NDWI 5p | EVI 50p | SVVI 75p |  | DVI 10p |
| Woodland 250m | EVI 90p | TCW 90p |  | TCB 75p |
| Grassland 300m | MNDWI 10p | TCB 25p |  | TCW 10p |
| NDWI 10p range | MNDWI 5p | SVVI 25p range |  | SVVI 10p range |
|  | Woodland 200m | NDVI 75p |  | TCB 50p |
|  | Woodland 150m | TCB 50p |  | SVVI 75p |
|  | Grassland 150m | TCB 10p range |  | EVI 90p |
|  | Arable 50m | TCW 10p |  | TCB 25p |
|  | MNDWI 75p | SVVI 90p |  | Woodland 250m |
|  | NDVI 90p | TCW 10p range |  | MNDWI 50p |
|  |  | TCG 10p |  | SVVI 25p range |
|  |  | SVVI 10p range |  | SVVI 90p |

TCB = Tassled Cap Brightness, TCG = Tassled Cap Greeness, TCW = Tassled Cap Wetness, EVI = Enhanced Vegetation Index, NDWI = Normalised Difference Water Index, NDVI = Normalised Difference Vegetation Index, GRVI = Green Red Vegetation Index, MNDWI = Modified Normalised Difference Water Index, DVI = Difference Vegetation Index, TVI = Triangular Vegetation Index, SVVI = Spectral Variability Vegetation Index, SAVI = Soil Adjusted Vegetation Index, 5p = 5th percentile, 10p = 10th percentile, 25p = 25th percentile, 50p = 50th percentile, 75p = 75th percentile, 90p = 90th percentile, 95p - 95th percentile. Variables are displayed in order of decreasing importance as determined by the random forest variable importance rankings, with the most important variable at the top.
