# Supplementary material for: Mapping small mammal optimal habitats using satellite-derived proxy variables and species distribution models
Source: PLoS One. 2023 Aug 17;18(8):e0289209. doi: 10.1371/journal.pone.0289209 (PMC10434852; doi:10.1371/journal.pone.0289209)
Supplement: S6 Table — (DOCX) [file pone.0289209.s006.docx]

**S6 Table. Remote sensing variables identified by the boruta feature selection analysis as important for each small mammal species for the Narati transect data.**

| **Transect** |  |
| --- | --- |
| *E. tancrei* | *M. baibacina* |
| NDVI 50p | Grassland 400m |
| Elevation | Woodland 500m |
| EVI 50p | TCB 10p |
| TCG 50p | TCB 25p |
| NDWI 90p range | TCB 50p |
| NDWI 90p | Grassland 350m |
| TCW 50p | Grassland 500m |
| SAVI 90p range | SVVI 75p |
| SVVI 50p | TCW 10p |
| TVI 50p | Slope |
| GRVI 90p | MNDWI 5p |
| DVI 50p | TCW 25p |
| NDVI 90p range | EVI 5p |
| DVI p90 | NDVI 50p |
| EVI 90p | TCW 50p |
| MNDWI 75p range | EVI 50p |
| SAVI 90p |  |
| NDVI 75p |  |
| TVI 90p |  |
| TCW 90p |  |
| NDWI 10p |  |
| Grassland 500m |  |
| NDVI 90p |  |
| EVI 5p |  |
| NDVI 95p |  |
| GRVI 10p |  |

TCB = Tassled Cap Brightness, TCW = Tassled Cap Wetness, TCG = Tassled Cap Greeness, EVI = Enhanced Vegetation Index, NDWI = Normalised Difference Water Index, NDVI = Normalised Difference Vegetation Index, GRVI = Green Red Vegetation Index, MNDWI = Modified Normalised Difference Water Index, DVI = Difference Vegetation Index, TVI = Triangular Vegetation Index, SVVI = Spectral Variability Vegetation Index, SAVI = Soil Adjusted Vegetation Index, 5p = 5th percentile, 10p = 10th percentile, 25p = 25th percentile, 50p = 50th percentile, 75p = 75th percentile, 90p = 90th percentile, 95p - 95th percentile. Variables are displayed in order of decreasing importance as determined by the random forest variable importance rankings, with the most important variable at the top.
