# Supplementary material for: Mapping small mammal optimal habitats using satellite-derived proxy variables and species distribution models
Source: PLoS One. 2023 Aug 17;18(8):e0289209. doi: 10.1371/journal.pone.0289209 (PMC10434852; doi:10.1371/journal.pone.0289209)
Supplement: S7 Table — (DOCX) [file pone.0289209.s007.docx]

**S7 Table. Remote sensing variables identified by the boruta feature selection analysis as important for each small mammal species for Sary Mogul.**

| **Trapline** |  | **Transect** |  |
| --- | --- | --- | --- |
| ***M. gregalis*** | ***C. migratorius*** | ***E. tancrei*** | ***M. gregalis*** |
| TCB 25P | NDWI 25p range | TCW 10p | TVI 50p |
| TCB 50P | NDWI 10P | DVI 25p range | DVI 50p |
| Grassland 250m | TVI 75P | TCG 25p range | NDWI 50p |
| Grassland 300m | GRVI 25p range | SAVI 25p range |  |
| Grassland 500m | NDWI 75P | NDVI 25p range |  |
| Grassland 450m | TVI 25p range | NDVI 5p range |  |
| Grassland 400m |  | EVI 90p |  |
| Grassland 200m |  |  |  |
| Grassland 350m |  |  |  |
| Grassland 150m |  |  |  |
| Grassland 100m |  |  |  |
| TCB 75P |  |  |  |
| TCW 10P |  |  |  |

TCB = Tassled Cap Brightness, TCW = Tassled Cap Wetness, TCG = Tassled Cap Greeness, EVI = Enhanced Vegetation Index, NDVI = Normalised Difference Vegetation Index, DVI = Difference Vegetation Index, SAVI = Soil Adjusted Vegetation Index, TVI = Triangular Vegetation Index, GRVI = Green Red Vegetation Index, NDWI = Normalised Difference Water Index, , 5p = 5th percentile, 10p = 10th percentile, 25p = 25th percentile, 50p = 50th percentile, 75p = 75th percentile, 90p = 90th percentile. Variables are displayed in order of decreasing importance as determined by the random forest variable importance rankings, with most important variable at the top.
