# Supplementary material for: Mapping small mammal optimal habitats using satellite-derived proxy variables and species distribution models
Source: PLoS One. 2023 Aug 17;18(8):e0289209. doi: 10.1371/journal.pone.0289209 (PMC10434852; doi:10.1371/journal.pone.0289209)
Supplement: S8 Table — n = number of trees, MLP = minimum leaf population, MN = maximum nodes, VPS = variables per split, BF = bag fraction. (DOCX) [file pone.0289209.s008.docx]

**S8 Table. Random Forest hyperparameter tuning results for the Narati trapline data****, displaying R^2^ values between predicted and observed values using leave-one-out cross validation.** n = number of trees, MLP = minimum leaf population, MN = maximum nodes, VPS = variables per split, BF = bag fraction.

| **Variables** | ***A. uralensis*** | ***M. obscurus*** | ***M. centralis*** | ***S. tianshanica*** | ***S. asper*** |
| --- | --- | --- | --- | --- | --- |
| n=200, MLP=2, MN=null, VPS=null, BF=5 | 0.737 | 0.860 | 0.819 | 0.644 | 0.730 |
| n=200, MLP=3, MN=null, VPS=null, BF=5 | 0.683 | 0.798 | 0.755 | 0.512 | 0.626 |
| n=200, MLP=4, MN=null, VPS=null, BF=5 | 0.637 | 0.748 | 0.703 | 0.463 | 0.583 |
| n=200, MLP=5, MN=null, VPS=null, BF=5 | 0.605 | 0.677 | 0.671 | 0.422 | 0.559 |
| n=200, MLP=6, MN=null, VPS=null, BF=5 | 0.585 | 0.619 | 0.647 | 0.400 | 0.509 |
| n=200, MLP=7, MN=null, VPS=null, BF=5 | 0.562 | 0.570 | 0.622 | 0.376 | 0.443 |
| n=200, MLP=8, MN=null, VPS=null, BF=5 | 0.453 | 0.519 | 0.605 | 0.325 | 0.374 |
| n=200, MLP=9, MN=null, VPS=null, BF=5 | 0.428 | 0.480 | 0.598 | 0.321 | 0.000 |
| n=200, MLP=10, MN=null, VPS=null, BF=5 | 0.417 | 0.452 | 0.582 | 0.310 | 0.000 |
| n=1, MLP=1, MN=null, VPS=null, BF=5 | 0.339 | 0.525 | 0.791 | 0.395 | 0.290 |
| n=2, MLP=1, MN=null, VPS=null, BF=5 | 0.521 | 0.722 | 0.773 | 0.406 | 0.600 |
| n=3, MLP=1, MN=null, VPS=null, BF=5 | 0.562 | 0.699 | 0.778 | 0.447 | 0.684 |
| n=4, MLP=1, MN=null, VPS=null, BF=5 | 0.557 | 0.764 | 0.784 | 0.474 | 0.663 |
| n=5, MLP=1, MN=null, VPS=null, BF=5 | 0.582 | 0.796 | 0.798 | 0.464 | 0.692 |
| n=6, MLP=1, MN=null, VPS=null, BF=5 | 0.610 | 0.821 | 0.771 | 0.501 | 0.718 |
| n=7, MLP=1, MN=null, VPS=null, BF=5 | 0.628 | 0.828 | 0.779 | 0.544 | 0.715 |
| n=8, MLP=1, MN=null, VPS=null, BF=5 | 0.651 | 0.819 | 0.805 | 0.534 | 0.748 |
| n=9, MLP=1, MN=null, VPS=null, BF=5 | 0.648 | 0.830 | 0.788 | 0.531 | 0.732 |
| n=10, MLP=1, MN=null, VPS=null, BF=5 | 0.655 | 0.847 | 0.777 | 0.559 | 0.728 |
| n=50, MLP=1, MN=null, VPS=null, BF=5 | 0.744 | 0.832 | 0.813 | 0.660 | 0.720 |
| n=100, MLP=1, MN=null, VPS=null, BF=5 | 0.747 | 0.843 | 0.833 | 0.643 | 0.721 |
| n=300, MLP=1, MN=null, VPS=null, BF=5 | 0.729 | 0.858 | 0.828 | 0.651 | 0.743 |
| n=500, MLP=1, MN=null, VPS=null, BF=5 | 0.731 | 0.856 | 0.825 | 0.663 | 0.742 |
| n=200, MLP=1, MN=2, VPS=null, BF=5 | 0.558 | 0.620 | 0.660 | 0.375 | 0.585 |
| n=200, MLP=1, MN=5, VPS=null, BF=5 | 0.669 | 0.783 | 0.771 | 0.608 | 0.728 |
| n=200, MLP=1, MN=10, VPS=null, BF=5 | 0.736 | 0.859 | 0.819 | 0.644 | 0.730 |
| n=200, MLP=1, MN=20, VPS=null, BF=5 | 0.737 | 0.860 | 0.819 | 0.644 | 0.730 |
| n=200, MLP=1, MN=30, VPS=null, BF=5 | 0.737 | 0.860 | 0.819 | 0.644 | 0.730 |
| n=200, MLP=1, MN=40, VPS=null, BF=5 | 0.737 | 0.860 | 0.819 | 0.644 | 0.730 |
| n=200, MLP=1, MN=50, VPS=null, BF=5 | 0.737 | 0.860 | 0.819 | 0.644 | 0.730 |
| n=200, MLP=1, MN=null, VPS=2, BF=5 | 0.719 | 0.841 | 0.801 | 0.644 | 0.720 |
| n=200, MLP=1, MN=null, VPS=3, BF=5 | 0.737 | 0.850 | 0.807 | 0.650 | 0.726 |
| n=200, MLP=1, MN=null, VPS=4, BF=5 | 0.747 | 0.860 | 0.819 | 0.658 | 0.730 |
| n=200, MLP=1, MN=null, VPS=5, BF=5 | 0.755 | 0.854 | 0.828 | 0.663 | 0.732 |
| n=200, MLP=1, MN=null, VPS=6, BF=5 | 0.753 | 0.853 | 0.831 | 0.669 | 0.730 |
| n=200, MLP=1, MN=null, VPS=7, BF=5 | 0.755 | 0.854 | 0.838 | 0.663 | 0.732 |
| n=200, MLP=1, MN=null, VPS=8, BF=5 | 0.753 | 0.856 | 0.842 | 0.657 | 0.733 |
| n=200, MLP=1, MN=null, VPS=9, BF=5 | 0.755 | 0.860 | 0.845 |  | 0.734 |
| n=200, MLP=1, MN=null, VPS=10, BF=5 | 0.754 | 0.860 | 0.846 |  | 0.737 |
| n=200, MLP=1, MN=null, VPS=11, BF=5 | 0.754 | 0.862 | 0.851 |  | 0.737 |
| n=200, MLP=1, MN=null, VPS=12, BF=5 | 0.753 | 0.861 | 0.851 |  | 0.735 |
| n=200, MLP=1, MN=null, VPS=13, BF=5 | 0.749 | 0.860 | 0.852 |  | 0.739 |
| n=200, MLP=1, MN=null, VPS=14, BF=5 | 0.750 | 0.861 | 0.854 |  | 0.739 |
| n=200, MLP=1, MN=null, VPS=15, BF=5 | 0.747 | 0.862 | 0.856 |  | 0.741 |
| n=200, MLP=1, MN=null, VPS=16, BF=6 |  | 0.860 | 0.855 |  | 0.742 |
| n=200, MLP=1, MN=null, VPS=17, BF=6 |  | 0.858 | 0.856 |  | 0.744 |
| n=200, MLP=1, MN=null, VPS=18, BF=6 |  | 0.860 | 0.856 |  | 0.744 |
| n=200, MLP=1, MN=null, VPS=19, BF=7 |  | 0.860 | 0.855 |  | 0.742 |
| n=200, MLP=1, MN=null, VPS=20, BF=7 |  | 0.859 | 0.855 |  | 0.745 |
| n=200, MLP=1, MN=null, VPS=21, BF=7 |  | 0.859 | 0.855 |  | 0.744 |
| n=200, MLP=1, MN=null, VPS=22, BF=8 |  |  | 0.854 |  | 0.745 |
| n=200, MLP=1, MN=null, VPS=23, BF=9 |  |  | 0.858 |  | 0.748 |
| n=200, MLP=1, MN=null, VPS=null, BF=1 | 0.375 | 0.499 | 0.543 | 0.249 | 0.000 |
| n=200, MLP=1, MN=null, VPS=null, BF=2 | 0.597 | 0.647 | 0.638 | 0.393 | 0.541 |
| n=200, MLP=1, MN=null, VPS=null, BF=3 | 0.659 | 0.739 | 0.698 | 0.495 | 0.605 |
| n=200, MLP=1, MN=null, VPS=null, BF=4 | 0.685 | 0.803 | 0.758 | 0.550 | 0.656 |
| n=200, MLP=1, MN=null, VPS=null, BF=5 | 0.737 | 0.860 | 0.819 | 0.644 | 0.730 |
| n=200, MLP=1, MN=null, VPS=null, BF=6 | 0.767 | 0.885 | 0.861 | 0.722 | 0.792 |
| n=200, MLP=1, MN=null, VPS=null, BF=7 | 0.780 | 0.907 | 0.886 | 0.771 | 0.835 |
| n=200, MLP=1, MN=null, VPS=null, BF=8 | 0.765 | 0.888 | 0.859 | 0.735 | 0.811 |
| n=200, MLP=1, MN=null, VPS=null, BF=9 | 0.761 | 0.884 | 0.854 | 0.733 | 0.801 |
