# Supplementary material for: Mapping small mammal optimal habitats using satellite-derived proxy variables and species distribution models
Source: PLoS One. 2023 Aug 17;18(8):e0289209. doi: 10.1371/journal.pone.0289209 (PMC10434852; doi:10.1371/journal.pone.0289209)
Supplement: S9 Table — n = number of trees, MLP = minimum leaf population, MN = maximum nodes, VPS = variables per split, BF = bag fraction. (DOCX) [file pone.0289209.s009.docx]

**S9 Table. Random Forest hyperparameter tuning results for the Narati transect data, displaying R^2^ values between predicted and observed values using leave-one-out cross validation.** n = number of trees, MLP = minimum leaf population, MN = maximum nodes, VPS = variables per split, BF = bag fraction.

| **Variables** | ***E. tancrei*** | ***M. baibacina*** |
| --- | --- | --- |
| n=200, MLP=2, MN=null, VPS=null, BF=5 | 0.856 | 0.764 |
| n=200, MLP=3, MN=null, VPS=null, BF=5 | 0.784 | 0.660 |
| n=200, MLP=4, MN=null, VPS=null, BF=5 | 0.726 | 0.595 |
| n=200, MLP=5, MN=null, VPS=null, BF=5 | 0.685 | 0.535 |
| n=200, MLP=6, MN=null, VPS=null, BF=5 | 0.644 | 0.473 |
| n=200, MLP=7, MN=null, VPS=null, BF=5 | 0.608 | 0.428 |
| n=200, MLP=8, MN=null, VPS=null, BF=5 | 0.574 | 0.387 |
| n=200, MLP=9, MN=null, VPS=null, BF=5 | 0.554 | 0.354 |
| n=200, MLP=10, MN=null, VPS=null, BF=5 | 0.528 | 0.322 |
| n=1, MLP=1, MN=null, VPS=null, BF=5 | 0.390 | 0.188 |
| n=2, MLP=1, MN=null, VPS=null, BF=5 | 0.479 | 0.324 |
| n=3, MLP=1, MN=null, VPS=null, BF=5 | 0.595 | 0.391 |
| n=4, MLP=1, MN=null, VPS=null, BF=5 | 0.685 | 0.532 |
| n=5, MLP=1, MN=null, VPS=null, BF=5 | 0.682 | 0.570 |
| n=6, MLP=1, MN=null, VPS=null, BF=5 | 0.724 | 0.582 |
| n=7, MLP=1, MN=null, VPS=null, BF=5 | 0.738 | 0.591 |
| n=8, MLP=1, MN=null, VPS=null, BF=5 | 0.766 | 0.597 |
| n=9, MLP=1, MN=null, VPS=null, BF=5 | 0.783 | 0.595 |
| n=10, MLP=1, MN=null, VPS=null, BF=5 | 0.790 | 0.612 |
| n=50, MLP=1, MN=null, VPS=null, BF=5 | 0.840 | 0.736 |
| n=100, MLP=1, MN=null, VPS=null, BF=5 | 0.851 | 0.756 |
| n=300, MLP=1, MN=null, VPS=null, BF=5 | 0.862 | 0.773 |
| n=500, MLP=1, MN=null, VPS=null, BF=5 | 0.867 | 0.777 |
| n=200, MLP=1, MN=2, VPS=null, BF=5 | 0.240 | 0.253 |
| n=200, MLP=1, MN=5, VPS=null, BF=5 | 0.722 | 0.564 |
| n=200, MLP=1, MN=10, VPS=null, BF=5 | 0.813 | 0.718 |
| n=200, MLP=1, MN=20, VPS=null, BF=5 | 0.853 | 0.764 |
| n=200, MLP=1, MN=30, VPS=null, BF=5 | 0.856 | 0.764 |
| n=200, MLP=1, MN=40, VPS=null, BF=5 | 0.856 | 0.764 |
| n=200, MLP=1, MN=50, VPS=null, BF=5 | 0.856 | 0.764 |
| n=200, MLP=1, MN=null, VPS=2, BF=5 | 0.841 | 0.784 |
| n=200, MLP=1, MN=null, VPS=3, BF=5 | 0.851 | 0.771 |
| n=200, MLP=1, MN=null, VPS=4, BF=5 | 0.852 | 0.764 |
| n=200, MLP=1, MN=null, VPS=5, BF=5 | 0.856 | 0.765 |
| n=200, MLP=1, MN=null, VPS=6, BF=5 | 0.859 | 0.768 |
| n=200, MLP=1, MN=null, VPS=7, BF=5 | 0.856 | 0.763 |
| n=200, MLP=1, MN=null, VPS=8, BF=5 | 0.861 | 0.758 |
| n=200, MLP=1, MN=null, VPS=9, BF=5 | 0.864 | 0.759 |
| n=200, MLP=1, MN=null, VPS=10, BF=5 | 0.860 | 0.755 |
| n=200, MLP=1, MN=null, VPS=11, BF=5 | 0.861 | 0.754 |
| n=200, MLP=1, MN=null, VPS=12, BF=5 | 0.852 | 0.757 |
| n=200, MLP=1, MN=null, VPS=13, BF=5 | 0.853 | 0.757 |
| n=200, MLP=1, MN=null, VPS=14, BF=5 | 0.854 | 0.759 |
| n=200, MLP=1, MN=null, VPS=15, BF=5 | 0.852 | 0.762 |
| n=200, MLP=1, MN=null, VPS=16, BF=6 | 0.851 | 0.760 |
| n=200, MLP=1, MN=null, VPS=17, BF=6 | 0.850 | 0.752 |
| n=200, MLP=1, MN=null, VPS=18, BF=6 | 0.850 |  |
| n=200, MLP=1, MN=null, VPS=19, BF=7 | 0.848 |  |
| n=200, MLP=1, MN=null, VPS=20, BF=7 | 0.844 |  |
| n=200, MLP=1, MN=null, VPS=21, BF=7 | 0.848 |  |
| n=200, MLP=1, MN=null, VPS=22, BF=8 | 0.847 |  |
| n=200, MLP=1, MN=null, VPS=23, BF=9 | 0.842 |  |
| n=200, MLP=1, MN=null, VPS=24, BF=9 | 0.840 |  |
| n=200, MLP=1, MN=null, VPS=25, BF=9 | 0.837 |  |
| n=200, MLP=1, MN=null, VPS=26, BF=9 | 0.839 |  |
| n=200, MLP=1, MN=null, VPS=null, BF=1 | 0.397 | 0.223 |
| n=200, MLP=1, MN=null, VPS=null, BF=2 | 0.566 | 0.446 |
| n=200, MLP=1, MN=null, VPS=null, BF=3 | 0.692 | 0.600 |
| n=200, MLP=1, MN=null, VPS=null, BF=4 | 0.789 | 0.693 |
| n=200, MLP=1, MN=null, VPS=null, BF=5 | 0.856 | 0.764 |
| n=200, MLP=1, MN=null, VPS=null, BF=6 | 0.903 | 0.833 |
| n=200, MLP=1, MN=null, VPS=null, BF=7 | 0.939 | 0.878 |
| n=200, MLP=1, MN=null, VPS=null, BF=8 | 0.905 | 0.844 |
| n=200, MLP=1, MN=null, VPS=null, BF=9 | 0.901 | 0.834 |
