# Supplementary material for: Mapping small mammal optimal habitats using satellite-derived proxy variables and species distribution models
Source: PLoS One. 2023 Aug 17;18(8):e0289209. doi: 10.1371/journal.pone.0289209 (PMC10434852; doi:10.1371/journal.pone.0289209)
Supplement: S10 Table — n = number of trees, MLP = minimum leaf population, MN = maximum nodes, VPS = variables per split, BF = bag fraction. (DOCX) [file pone.0289209.s010.docx]

**S10 Table. Random Forest hyperparameter tuning results for Sary Mogul, displaying R^2^ values between predicted and observed values using leave-one-out cross validation.** n = number of trees, MLP = minimum leaf population, MN = maximum nodes, VPS = variables per split, BF = bag fraction.

|  | **Trapline** | | **Transect** | |
| --- | --- | --- | --- | --- |
| **Variables** | ***C. migratorius*** | ***M. gregalis*** | ***E. tancrei*** | ***M. gregalis*** |
| n=200, MLP=2, MN=null, VPS=null, BF=5 | 0.711 | 0.666 | 0.685 | 0.495 |
| n=200, MLP=3, MN=null, VPS=null, BF=5 | 0.539 | 0.459 | 0.486 | 0.363 |
| n=200, MLP=4, MN=null, VPS=null, BF=5 | 0.430 | 0.337 | 0.407 | 0.288 |
| n=200, MLP=5, MN=null, VPS=null, BF=5 | 0.351 | 0.314 | 0.373 | 0.232 |
| n=200, MLP=6, MN=null, VPS=null, BF=5 | 0.292 | 0.296 | 0.336 | 0.180 |
| n=200, MLP=7, MN=null, VPS=null, BF=5 | 0.241 | 0.268 | 0.308 | 0.150 |
| n=200, MLP=8, MN=null, VPS=null, BF=5 | 0.183 | 0.235 | 0.264 | 0.139 |
| n=200, MLP=9, MN=null, VPS=null, BF=5 | 0.144 | 0.209 | 0.221 | 0.124 |
| n=200, MLP=10, MN=null, VPS=null, BF=5 | 0.127 | 0.194 | 0.000 | 0.000 |
| n=1, MLP=1, MN=null, VPS=null, BF=5 | 0.126 | 0.309 | 0.020 | 0.362 |
| n=2, MLP=1, MN=null, VPS=null, BF=5 | 0.214 | 0.323 | 0.279 | 0.363 |
| n=3, MLP=1, MN=null, VPS=null, BF=5 | 0.299 | 0.551 | 0.281 | 0.402 |
| n=4, MLP=1, MN=null, VPS=null, BF=5 | 0.435 | 0.550 | 0.577 | 0.382 |
| n=5, MLP=1, MN=null, VPS=null, BF=5 | 0.488 | 0.626 | 0.577 | 0.399 |
| n=6, MLP=1, MN=null, VPS=null, BF=5 | 0.476 | 0.634 | 0.682 | 0.371 |
| n=7, MLP=1, MN=null, VPS=null, BF=5 | 0.544 | 0.626 | 0.719 | 0.369 |
| n=8, MLP=1, MN=null, VPS=null, BF=5 | 0.556 | 0.630 | 0.719 | 0.375 |
| n=9, MLP=1, MN=null, VPS=null, BF=5 | 0.572 | 0.635 | 0.677 | 0.389 |
| n=10, MLP=1, MN=null, VPS=null, BF=5 | 0.577 | 0.625 | 0.648 | 0.386 |
| n=50, MLP=1, MN=null, VPS=null, BF=5 | 0.708 | 0.681 | 0.666 | 0.448 |
| n=100, MLP=1, MN=null, VPS=null, BF=5 | 0.703 | 0.644 | 0.693 | 0.466 |
| n=300, MLP=1, MN=null, VPS=null, BF=5 | 0.706 | 0.670 | 0.696 | 0.507 |
| n=500, MLP=1, MN=null, VPS=null, BF=5 | 0.711 | 0.671 | 0.697 | 0.505 |
| n=200, MLP=1, MN=2, VPS=null, BF=5 | 0.162 | 0.535 | 0.466 | 0.155 |
| n=200, MLP=1, MN=5, VPS=null, BF=5 | 0.580 | 0.659 | 0.684 | 0.463 |
| n=200, MLP=1, MN=10, VPS=null, BF=5 | 0.711 | 0.666 | 0.685 | 0.495 |
| n=200, MLP=1, MN=20, VPS=null, BF=5 | 0.711 | 0.666 | 0.685 | 0.495 |
| n=200, MLP=1, MN=30, VPS=null, BF=5 | 0.711 | 0.666 | 0.685 | 0.495 |
| n=200, MLP=1, MN=40, VPS=null, BF=5 | 0.711 | 0.666 | 0.685 | 0.495 |
| n=200, MLP=1, MN=50, VPS=null, BF=5 | 0.711 | 0.666 | 0.685 | 0.495 |
| n=200, MLP=1, MN=null, VPS=2, BF=5 | 0.711 | 0.676 | 0.685 | 0.543 |
| n=200, MLP=1, MN=null, VPS=3, BF=5 | 0.707 | 0.666 | 0.719 | 0.576 |
| n=200, MLP=1, MN=null, VPS=4, BF=5 | 0.711 | 0.660 | 0.752 |  |
| n=200, MLP=1, MN=null, VPS=5, BF=5 | 0.705 | 0.660 | 0.765 |  |
| n=200, MLP=1, MN=null, VPS=6, BF=5 | 0.705 | 0.659 | 0.767 |  |
| n=200, MLP=1, MN=null, VPS=7, BF=5 |  | 0.662 | 0.774 |  |
| n=200, MLP=1, MN=null, VPS=8, BF=5 |  | 0.667 |  |  |
| n=200, MLP=1, MN=null, VPS=9, BF=5 |  | 0.667 |  |  |
| n=200, MLP=1, MN=null, VPS=10, BF=5 |  | 0.660 |  |  |
| n=200, MLP=1, MN=null, VPS=11, BF=5 |  | 0.656 |  |  |
| n=200, MLP=1, MN=null, VPS=12, BF=5 |  | 0.656 |  |  |
| n=200, MLP=1, MN=null, VPS=13, BF=5 |  | 0.661 |  |  |
| n=200, MLP=1, MN=null, VPS=null, BF=1 | 0.124 | 0.276 | 0.261 | 0.108 |
| n=200, MLP=1, MN=null, VPS=null, BF=2 | 0.309 | 0.351 | 0.381 | 0.208 |
| n=200, MLP=1, MN=null, VPS=null, BF=3 | 0.441 | 0.422 | 0.434 | 0.299 |
| n=200, MLP=1, MN=null, VPS=null, BF=4 | 0.604 | 0.531 | 0.577 | 0.407 |
| n=200, MLP=1, MN=null, VPS=null, BF=5 | 0.711 | 0.666 | 0.685 | 0.495 |
| n=200, MLP=1, MN=null, VPS=null, BF=6 | 0.774 | 0.733 | 0.779 | 0.590 |
| n=200, MLP=1, MN=null, VPS=null, BF=7 | 0.830 | 0.775 | 0.810 | 0.670 |
| n=200, MLP=1, MN=null, VPS=null, BF=8 | 0.814 | 0.763 | 0.795 | 0.661 |
| n=200, MLP=1, MN=null, VPS=null, BF=9 | 0.815 | 0.761 | 0.792 | 0.656 |
